# Cold-induced anthocyanin regulation in horticultural crops: mechanistic basis, trade-offs, and decision-driven temperature management in plant factories

**DOI:** 10.1093/hr/uhag205

**Published:** 2026-06-11

**Authors:** Hadiqa Anum, Ying Yang, Nazim S Gruda, Lei Wang, Songheng Jin

**Affiliations:** Jiyang College, Zhejiang A&F University, Zhuji 311800, China; Jiyang College, Zhejiang A&F University, Zhuji 311800, China; Department of Horticulture, University of Bonn, Bonn 53113, Germany; Jiyang College, Zhejiang A&F University, Zhuji 311800, China; Jiyang College, Zhejiang A&F University, Zhuji 311800, China

## Abstract

With the expansion of modern horticultural production systems, anthocyanins have attracted increasing attention for their dual role in imparting vibrant coloration and delivering health-promoting benefits. Preharvest temperature management, particularly controlled low-temperature exposure, has emerged as an effective strategy to enhance anthocyanin accumulation in horticultural crops. Despite extensive molecular insights, decision-driven temperature management (DDTM) frameworks that integrate species-, genotype-, and operational parameter-specific strategies for plant factory applications remain lacking. This review addresses this gap by proposing a DDTM-based framework that links molecular regulation to operational protocols for precise anthocyanin enrichment. Evidence is synthesized across diverse horticultural crops representing major production families, including Brassicaceae, Solanaceae, Rosaceae, and other representative horticultural groups, which were selected for their economic importance, high anthocyanin potential, and relevance to human health. Species- and genotype-dependent responses are highlighted, and key operational parameters, including temperature range, exposure duration, and preharvest timing, are analyzed to balance pigmentation intensity, biomass production, and energy efficiency. Plant factories are emphasized as enabling platforms for short-term end-of-cycle cooling and diurnal temperature modulation, which can be integrated with light spectrum management to optimize anthocyanin accumulation while maintaining yield stability. By organizing current knowledge into practical, decision-oriented temperature strategies, this review provides a framework to improve the nutritional quality, compositional consistency, and functional value of anthocyanin-rich crops, supporting horticulture-driven quality assurance and resilient controlled-environment production systems.

## Graphical Abstract

**Graphical Abstract:**
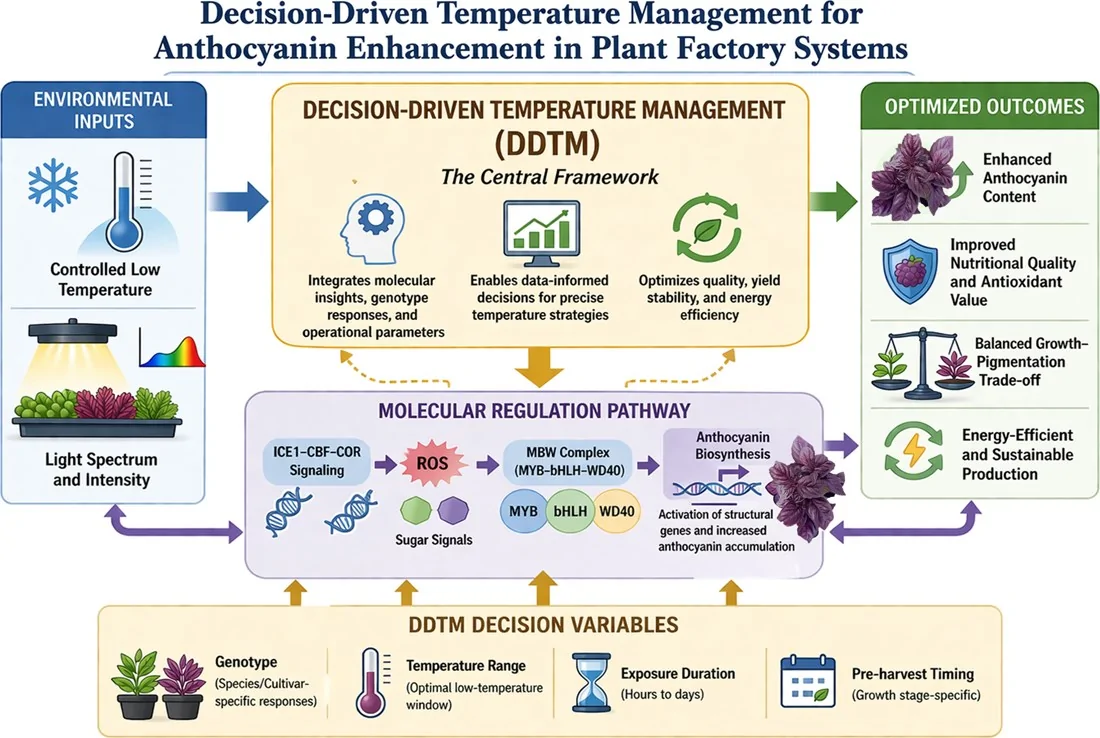

## Introduction

Growing consumer demand for fresh fruits and vegetables with appealing red, purple, and blue hues has intensified interest in horticultural crops [1, 2]. These colors, primarily driven by anthocyanins, are associated with perceived freshness, nutritional value, and premium quality, while also contributing to antioxidant capacity, cardiometabolic health, and the overall functional potential of food [1, 3]. In plants, anthocyanins play critical roles in UV protection, cold stress tolerance, and pollinator attraction, supporting growth and reproduction [3]. Temperature is a key regulator of anthocyanin biosynthesis: high temperatures often suppress pigment accumulation by destabilizing anthocyanins and downregulating biosynthetic genes, whereas low temperatures frequently stimulate pigmentation through transcriptional and metabolic reprogramming [4, 5]. For instance, red leaf lettuce and purple cauliflower have been shown to exhibit anthocyanin increases of 1.5–3.0 mg·g^−1^ FW under cold induction, making them ideal model crops for studying temperature-mediated pigmentation [6–8]. However, cold stress can also negatively affect plant growth and photosynthetic capacity, resulting in trade-offs between pigmentation and biomass accumulation, thereby making temperature management a complex but powerful tool for crop improvement [4, 9].

Controlled-environment agriculture (CEA) systems represent agricultural production systems in which environmental conditions such as temperature, light, CO₂, and humidity are partially or fully regulated to optimize plant growth and quality [10]. CEA includes a range of production systems, from greenhouses to highly controlled plant factories and vertical farming (VF) systems. Within this framework, plant factories represent advanced CEA systems that enable precise and independent regulation of environmental factors, thereby allowing fine-tuned manipulation of secondary metabolism, including anthocyanin accumulation, without compromising yield [11, 12]. Cold-induced anthocyanin enhancement is therefore an increasingly explored strategy for quality-oriented cultivation, with applications in end-of-cycle cooling, diurnal temperature modulation, and integration with light spectra [13]. Such interventions have been reported to improve anthocyanin content in targeted organs, such as leaves and petioles, highlighting the potential of precision cultivation [3]. Predictable control of anthocyanin content in such systems has important implications for food quality assurance, nutritional consistency, and functional food production [3, 14].

Despite growing research on cold-induced anthocyanin accumulation, critical gaps remain. Many existing reviews assume uniform responses across species, overlooking the variation among plant families such as Brassicaceae, Solanaceae, and Rosaceae [15, 16]. Mechanistic explanations are often simplified, with limited discussion of how transcriptional networks, reactive oxygen species (ROS), sugar signaling, and the MYB-bHLH-WD40 (MBW) regulatory complex interact under varying environmental and developmental contexts [4, 17]. Additionally, few studies consider plant factory constraints—such as energy costs, microclimate heterogeneity, and production-level trade-offs—which limits their real-world applicability [18, 19].

Addressing these limitations requires viewing cold-induced anthocyanin accumulation as a context-dependent process. Species-specific regulatory architecture, physiological trade-offs between growth and secondary metabolism, and system-level environmental constraints jointly determine pigment accumulation outcomes. Consequently, transcriptional pathways—such as ICE–CBF signaling, ROS-mediated redox regulation, sugar-driven carbon reallocation, and MBW complex plasticity—are considered together rather than in isolation.

Building on this perspective, this review is structured in a two-part framework: (i) the molecular mechanisms of temperature-induced anthocyanin regulation in horticultural plants and (ii) the precision regulation of anthocyanin accumulation within CEA, with a focus on VF systems. Within the VF section, a decision-driven temperature management (DDTM) approach is illustrated using case studies of red leaf lettuce [20] and purple cauliflower [21], including their operational parameters, anthocyanin outcomes, and energy considerations. By linking molecular regulation with actionable cultivation strategies, this framework provides a coherent strategy for maximizing anthocyanin content while maintaining crop productivity and system-level efficiency.

## Part 1: molecular mechanisms of temperature-induced anthocyanin regulation in horticultural plants

### Anthocyanins as quality determinants in controlled-environment crops

Anthocyanins function as adjustable quality traits in controlled-environment crop production, where they integrate chemical structure, biosynthetic regulation, environmental responsiveness, and nutritional relevance [10, 12, 22]. Their accumulation reflects coordinated control across molecular, transcriptional, and environmental layers [10, 23, 24], which determines pigment intensity, stability, and suitability for manipulation under engineered growth conditions (Fig. 1).

**Figure 1:**
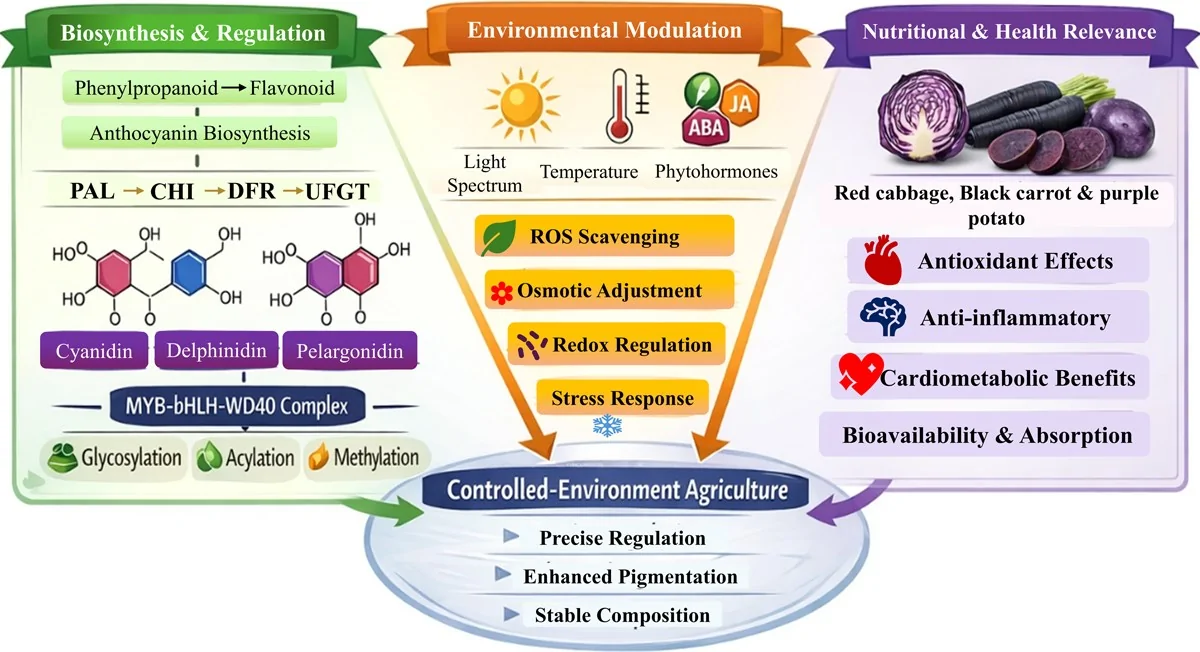
Integrated framework of temperature-regulated anthocyanin enhancement in controlled-environment crops. Anthocyanin biosynthesis is regulated by the MYB–bHLH–WD40 complex and influenced by temperature-driven redox, metabolic, and hormonal signals. In controlled environments, precise temperature management enables targeted anthocyanin accumulation, improving pigmentation, nutritional quality, and compositional stability of crops.

### Biosynthesis, structural diversity, and regulatory control

Anthocyanins originate from the phenylpropanoid-flavonoid pathway and are primarily derived from six anthocyanidins—cyanidin, delphinidin, pelargonidin, peonidin, petunidin, and malvidin—sharing a characteristic C6-C3-C6 flavylium-based structure responsible for pH-dependent coloration and redox activity [3, 4]. Structural diversification mediated by glycosylation, methylation, and acylation results in chemically stable anthocyanins that support species-specific pigmentation and enhanced degradation resistance, with acylated forms showing particular advantages for postharvest stability and shelf-life [25, 26]. Biosynthesis proceeds through phenylpropanoid, flavonoid, and late biosynthetic phases before vacuolar sequestration [4, 27]. Transcriptional regulation is primarily governed by the MBW complex, which coordinates the expression of early and late biosynthetic genes [3, 4]. Negative regulators, including R3-MYB proteins, modulate pathway activity by inhibiting MBW complex formation under noninductive conditions [4, 28]. Although this regulatory architecture is conserved, its relative importance compared to post-transcriptional and epigenetic regulation varies among species, contributing to differential environmental sensitivity [29, 30].

### Nutritional significance, bioavailability, and relevance to controlled environments

Anthocyanins are valued not only for pigmentation but also for their broad health-promoting effects, including antioxidant, anti-inflammatory, cardioprotective, and metabolic benefits [3]. These effects depend on both total anthocyanin content and molecular composition, which vary widely among crops [31]. High-anthocyanin vegetables—such as red cabbage, black carrot, and purple-fleshed tubers—highlight the nutritional potential of targeted enhancement strategies [4, 32]. Despite strong *in vitro* bioactivity, *in vivo* effects depend on stability, metabolism, and bioavailability [33, 34]. Structural features such as glycosylation and acylation influence gastrointestinal stability and absorption, while phenolic metabolites may contribute substantially to biological effects [4, 34].

CEA allows precise regulation of anthocyanin accumulation by minimizing environmental variability and aligning inputs with species- and cultivar-specific biosynthetic responses [3, 4]. This precision enables the predictable enhancement of pigment content and compositional quality, positioning anthocyanins as integrative quality indicators linking metabolic regulation, nutritional functionality, and production efficiency [35–37]. Together, anthocyanin composition, regulatory plasticity, and environmental sensitivity determine pigment quality and the feasibility of temperature- and light-based manipulation [3, 38], supporting evaluation as quality-driven, species-dependent traits rather than uniform stress markers.

### Environmental and hormonal modulation of anthocyanin accumulation

Anthocyanin biosynthesis is highly dynamic and regulated by environmental factors, particularly light and temperature, through coordinated transcriptional and hormonal signaling networks [39, 40]. Consequently, anthocyanin levels varies with species, genotype, developmental stage, and growth conditions rather than representing a uniform stress response [3]. Light quality, intensity, and photoperiod are major drivers of anthocyanin accumulation, acting through photoreceptors such as cryptochromes, UVR8, and phytochromes, which activate HY5 and R2R3-MYB transcription factors and converge on the MBW-regulated gene expression [4, 41, 42]. Blue and UV-A wavelengths are most effective in promoting anthocyanin accumulation, whereas red and far-red light modulate this response through phytochrome-mediated signaling [3, 4]. Genotype-specific responses are evident in Brassicaceae; for example, watercress cultivars exhibit differential anthocyanin and flavonol accumulation under identical spectral conditions due to genetic variation [43]. Dynamic light regulation, involving the temporal modulation of spectral composition, intensity, and duration, further enhances yield, nutritional quality, morphology, postharvest shelf-life, and energy-use efficiency compared to static lighting regimes [44]. In controlled environments, spectral tuning improves anthocyanin content, although outcomes depend strongly on genotype × light interactions and system-level energy efficiency [4, 37].

Temperature functions as both an independent regulator and a modulator of other environmental signals that influence anthocyanin accumulation [45]. As a standalone factor, low temperature activates ICE–CBF signaling and downstream transcriptional cascades that enhance MBW complex activity and structural gene expression. In addition, temperature interacts with light signaling pathways, where cold conditions enhance HY5 stability and photoreceptor-mediated transcription, thereby reinforcing anthocyanin biosynthesis [3, 21]. These interactions demonstrate that anthocyanin accumulation is governed by integrated environmental signaling networks rather than single-factor control.

Phytohormones provide an additional regulatory layer that links environmental cues with anthocyanin biosynthesis [41, 46]. Temperature-induced modulation of phytohormone biosynthesis and signaling pathways directly or indirectly regulates MBW complex activity and downstream structural genes [30, 41]. Abscisic acid and cytokinins generally promote anthocyanin accumulation, whereas gibberellins, auxin, ethylene, and jasmonic acid often suppress pigmentation by prioritizing growth-related processes [5, 30]. Together, these regulatory interactions ensure that anthocyanin accumulation remains environmentally responsive and developmentally coordinated.

## Molecular and physiological mechanisms of cold-induced anthocyanin biosynthesis

Cold-induced anthocyanin accumulation is a conditional, multi-layered process that emerges from the coordinated interaction of temperature perception, transcriptional regulation, redox signaling, and carbon metabolism [47]. The magnitude and timing of anthocyanin induction are highly context-dependent, shaped by species identity, developmental stage, chilling intensity, and the plant’s metabolic and physiological capacity [48, 49]. Moderate chilling can stimulate anthocyanin biosynthesis, thereby enhancing pigmentation and stress protection, whereas prolonged or extreme cold may suppress biosynthetic pathways, destabilize pigments, or reduce growth [39, 50]. These observations highlight that cold-induced anthocyanin accumulation is not a uniform stress response but a finely regulated, conditional adaptation strategy.

### Cold perception and CBF–COR cascades

Plant perception of low temperature initiates the ICE1–CBF–COR transcriptional cascade, a primary upstream regulatory module linking environmental cues to cold-responsive gene expression and anthocyanin biosynthesis [51, 52]. In this hierarchy, ICE1 functions as a cold-responsive transcription factor that activates CBF genes, which in turn regulate COR genes involved in cold acclimation and cellular homeostasis [53, 54]. These early transcriptional events establish a permissive physiological state that enables downstream metabolic reprogramming rather than directly inducing anthocyanin biosynthesis [3, 4]. Crosstalk between CBF signaling and flavonoid-related transcription factors enables integration of cold signals with secondary metabolism [54].

**Figure 2:**
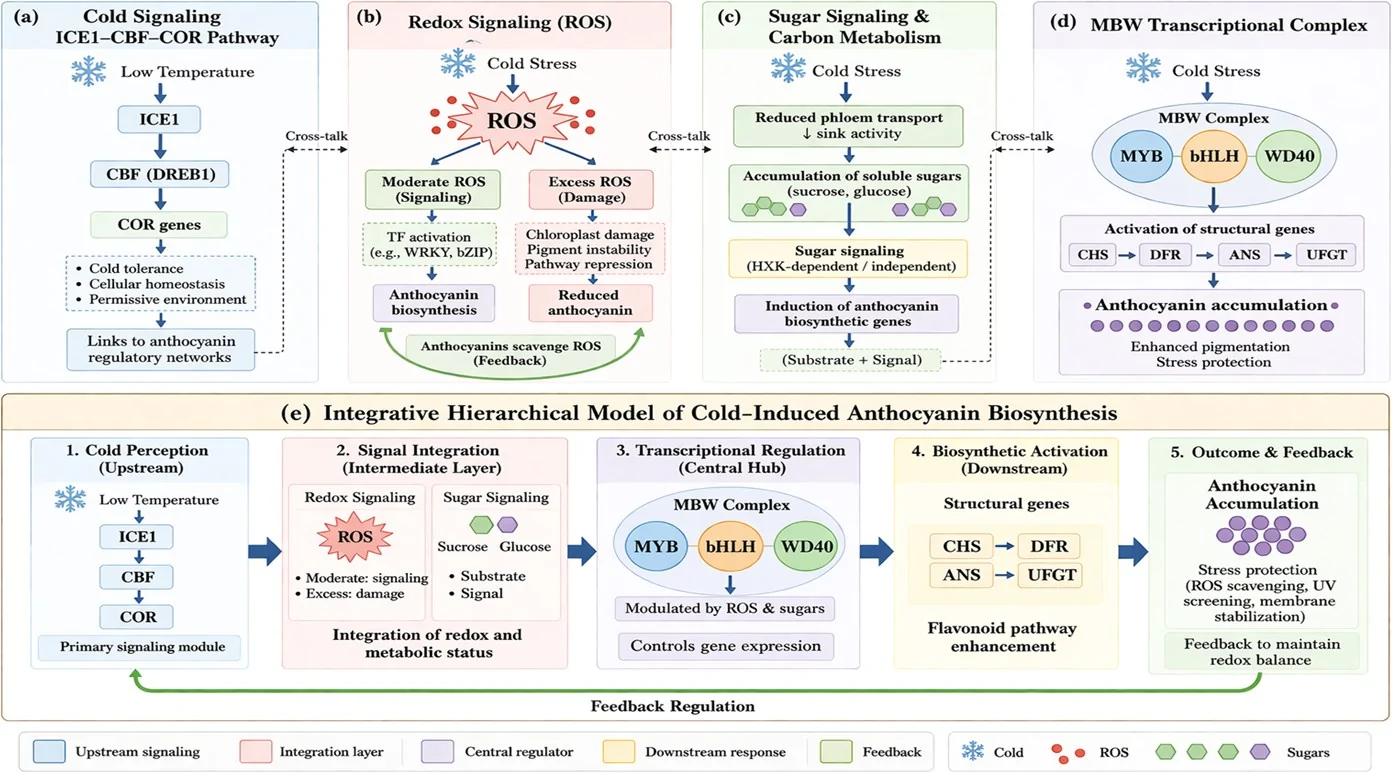
Cold-induced anthocyanin regulation. (a) Low temperature activates the ICE1–CBF–COR signaling pathway, establishing a permissive state for anthocyanin production. (b) Cold-induced ROS function as signaling molecules at moderate levels but cause damage at high levels, with anthocyanins contributing to ROS scavenging. (c) Sugar signaling integrates metabolic status, in which accumulated soluble sugars act as substrates and signals to induce anthocyanin biosynthesis. (d) MBW complex plasticity. These signals converge on the MBW (MYB–bHLH–WD40) transcriptional complex, which regulates structural genes (*CHS, DFR, ANS, UFGT*). (e) Integrative model illustrating the hierarchical regulation of cold-induced anthocyanin biosynthesis, linking upstream cold signaling (ICE1–CBF–COR), intermediate redox and sugar signaling, and downstream MBW-mediated transcriptional regulation.

Downstream of this cascade, cold-induced redox changes, particularly ROS accumulation, together with sugar signaling, act as intermediate regulatory layers integrating metabolic status with environmental cues [55]. These signals collectively modulate the activity of the MBW transcriptional complex, which serves as the central regulator of anthocyanin biosynthetic genes [56]. Crosstalk between CBF signaling and flavonoid-related transcription factors further reinforces the connection between cold perception and secondary metabolism [57]. To clarify the regulatory hierarchy, cold-induced anthocyanin biosynthesis follows a sequential framework in which the ICE1–CBF–COR cascade acts as the primary upstream signaling module, followed by the integration of redox and sugar signals that collectively regulate MBW complex activity and downstream anthocyanin biosynthetic gene expression (Fig. 2a–e, with panel a illustrating the ICE1–CBF–COR cascade framework).

### ROS: signaling vs damage

Cold stress disrupts cellular redox homeostasis, leading to ROS accumulation [4, 52]. At moderate levels, ROS act as signaling molecules that trigger stress-responsive transcription factors and secondary metabolite pathways, including anthocyanin biosynthesis [58]. Anthocyanins themselves function as ROS scavengers, forming a feedback loop that stabilizes redox status and prevents oxidative damage [40, 58]. However, excessive ROS generated during prolonged or extreme cold can damage chloroplasts, destabilize pigments, and suppress biosynthetic activity [4, 45]. This biphasic relationship explains why mild chilling promotes pigmentation, whereas extreme cold results in chlorosis, growth inhibition, and metabolic repression [4, 59] (Fig. 2b). The timing and intensity of ROS accumulation, together with species-specific sensitivity, define the effective thermal range for beneficial anthocyanin induction.

### Sugar accumulation and carbon allocation

Cold temperatures often reduce sink activity and phloem transport, causing soluble sugars such as sucrose and glucose to accumulate in source tissues [60]. These sugars serve both as substrates for anthocyanin biosynthesis and as signaling molecules that induce pathway genes through hexokinase-dependent and hexokinase-independent mechanisms [55]. The efficacy of sugar-mediated induction depends on photosynthetic activity and carbon availability (Fig. 2c). Under moderate cold, accumulated sugars are efficiently redirected into anthocyanin production, contributing to pigmentation and stress tolerance [4, 60]. Severe or prolonged chilling, however, often impairs photosynthesis, resulting in sugar accumulation that reflects metabolic repression rather than active biosynthesis [3, 61]. Subsequently, anthocyanin accumulation under cold stress frequently coincides with growth suppression or delayed development, highlighting a trade-off between secondary metabolite production and biomass accumulation.

### MBW complex plasticity

The MBW transcriptional complex serves as a central regulator of anthocyanin biosynthesis across plant species [62]. Cold stress modulates the expression and activity of MYB and bHLH components, thereby influencing metabolic flux through the biosynthetic pathway (Fig. 2d). Species-specific plasticity in MBW composition and activity determines the magnitude of anthocyanin induction [63]. Accordingly, cold-induced anthocyanin accumulation is highly species- and cultivar-dependent, reflecting variation in MBW transcription factors, cold signaling components, and structural genes [14]. Specific crops and their corresponding genes, regulatory elements, and biosynthetic pathways are summarized in Table 1, facilitating cross-species comparisons of cold-induced anthocyanin regulation. Moderate low-temperature exposure generally activates MBW regulators and downstream biosynthetic genes, promoting pigmentation; however, the presence of repressor MYBs or species-specific regulatory constraints may limit this response [4, 37]. To enhance practical applicability, future studies should focus on quantifying anthocyanin content alongside growth and yield trade-offs, temperature thresholds, exposure duration, and organ-specific pigmentation across representative cultivars. Target crops such as red-leaf lettuce, kale, purple cauliflower, tomato, and eggplant provide valuable model systems to establish genotype-specific optimization protocols for controlled-environment anthocyanin enhancement.

Collectively, the evidence supports a conditional response model in which cold-induced anthocyanin accumulation occurs only when: (i) cold signaling is active but not excessive, (ii) redox homeostasis remains within tolerable limits, (iii) sufficient carbon substrates are available, and (iv) MBW complexes are permissively regulated. Failure of any component (e.g. excessive ROS, carbon limitation, or repressive transcriptional regulation) results in diminished or absent pigmentation [9, 88]. This integrative interpretation accounts for the high variability observed across species, cultivars, and experimental conditions and highlights why cold treatments must be carefully tailored in plant factory systems to achieve predictable outcomes. An integrative hierarchical model summarizing these regulatory relationships is presented in Fig. 2e.

## Part 2: precision regulation of anthocyanins in vertical farming systems

### Plant factory systems as platforms for targeted temperature manipulation

Plant factories integrate CEA, automation, and food systems engineering to enable crop production largely independent of external climatic variability [89, 90]. Unlike greenhouses or growth chambers, these systems allow precise programming and reproducibility of temperature, light, humidity, and air composition across production cycles [11, 18]. This level of control is particularly relevant for anthocyanin accumulation, a temperature-sensitive quality trait that interacts strongly with light and carbon availability [3, 4]. Species- and developmental-stage-dependent responses highlight the need for targeted temperature strategies rather than uniform cooling.

**Table 1:** Genes and pathways underlying cold-induced anthocyanin accumulation in model plants and other crop species.

**Crop/species**	**Gene /gene (IDs)**	**Role/pathway**	**Cold-induced anthocyanin effect**	**References**
*Arabidopsis thaliana* (reference model)	*MYB75/PAP1* (AT1G56650)	MBW regulatory MYB TF	Strongly induced under moderate cold; directly activates CHS, F3H, DFR, ANS, and UFGT, leading to anthocyanin accumulation.	[64, 65]
	*CBF1* (AT4G25490), *CBF2* (AT4G25470), *CBF3* (AT4G25480)	ICE1–CBF–COR cold signaling	Indirectly promotes anthocyanin biosynthesis via cold-responsive transcriptional reprogramming	[56]
	*ICE1* (AT3G26744)	Cold perception TF	Upstream activator of the CBF cascade	[66]
	*CHS* (AT5G13930), *CHI* (AT3G55120), *F3H* (AT3G51240), *DFR* (AT5G42800), *ANS* (AT4G22880)	Structural anthocyanin biosynthesis genes	Cold-induced upregulation enhances anthocyanin biosynthesis	[21, 67, 68]
	*UGT79B1* (AT5G54060), *UGT75C1* (AT4G14090)	UDP-glycosyltransferases	Stabilizes anthocyanins via glycosylation	[69, 70]
*Brassica rapa* (Chinese cabbage)	*BrMYB2*	MBW activator	Cold-inducible MYB that activates structural genes (DFR, ANS)	[71]
	*BrTT8*	bHLH TF (MBW partner)	Interacts with MYB to form the MBW complex under cold condition	[72]
	*BrDFR* (Bra027457), *BrANS1* (Bra013652), *BrANS2* (Bra019350)	Structural anthocyanin genes	Cold-induced expression increases pigment accumulation	[20, 73]
	*BrUGT79B1.1* (Bra003021), *BrUGT79B1.2* (Bra035004)	Glycosylation enzymes	Supports pigment stabilization	[74]
*Malus domestica* (apple)	*MdMYB1, MdMYB10, MdMYB308L*	MBW regulatory MYBs	Cold enhances anthocyanin via transcriptional activation and post-translational stabilization (sumoylation via MdSIZ1)	[71]
	*MdbHLH3*	MBW partner	Synergistically activates anthocyanin biosynthetic genes with MYBs	[75]
	*MdDFR, MdANS, MdUFGT*	Structural genes	Cold-induced activation drives anthocyanin accumulation	[76]
*Fragaria × ananassa/F. vesca* (strawberry)	*FvMYB10*	MBW regulatory MYB	Cold-induced activation of anthocyanin biosynthetic pathway	[77, 78]
	*FvMAPK3*	MAP kinase	Enhances MYB10 activity via MAPK signaling under cold stress	[79]
*Vitis vinifera* (grape)	*VvCHS2, VvCHS3, VvF3H1*	Structural biosynthetic genes	Cold enhances early biosynthetic steps leading to anthocyanin accumulation	[67]
	*VvUFGT*	UDP-glucosyltransferase	Cold-responsive enzyme controlling final anthocyanin stabilization	[80]
*Solanum lycopersicum* (tomato)	HY5, bHLH TFs	MBW*/*CBF-independent regulation	Cold and light signals coordinately regulate anthocyanin accumulation	[57]
*Citrus sinensis* (blood orange)	*Ruby1*	MYB regulator	Cold night temperatures activate Ruby1, triggering strong peel anthocyanin accumulation	[81]
*Glycine max* (soybean)	*GmIMaT1, GmIMaT3*	Isoflavone biosynthesis	Cold enhances flavonoid biosynthesis contributing to stress tolerance	[82]
*Lactuca sativa* (lettuce)	*LsMYB, HY5*	MBW*/*light–cold integration	Cold and light synergistically induce anthocyanin biosynthesis with strong genotype-dependent variation	[6]
*Daucus carota* (black carrot)	*DcMYB6, DcMYB7*(DCAR_010757)	MYB regulators	High baseline anthocyanin; further enhanced under cold; stable acylated anthocyanins	[83, 84]
*Ipomoea batatas* (purple sweet potato)	IbMYB1 (AB576660)	Cold enhances storage root pigmentation	Cold enhances storage root pigmentation and anthocyanin stability	[85]
*Ocimum basilicum* (purple basil)	ObMYB, HY5	Light–temperature re regulation	Moderate cold enhances anthocyanin pigmentation via light–temperature interaction	[86]
*Raphanus sativus* (red radish)	RsMYB1 (ABF94624)	MYB-regulator	Cold induces anthocyanin accumulation in hypocotyl and root tissues	[87]

Beyond yield stability, plant factories function as platforms for nutritional standardization, food safety assurance, and resource-efficient production [14, 89]. Precise temperature regulation supports reproducible modulation of secondary metabolites and consistent nutritional quality in fresh and minimally processed foods [91], while enclosed systems reduce exposure to contaminants and pesticide residues, improving traceability and safety [92].

### Defining plant factories beyond growth chambers

Plant factories evolved from early controlled-environment systems and were first commercialized in Japan in the late 1970s [93, 94]. Their rapid global expansion reflects increasing demand for climate-resilient, safe, and resource-efficient food production [89, 93]. By relying on artificial lighting, hydroponic or aeroponic nutrient delivery, and centralized environmental control, plant factories enable year-round production independent of seasonal variation [3, 93, 95].

Compared with greenhouses, plant factories provide finer control over temperature, humidity, airflow, and light spectra, enabling reproducible manipulation of temperature-sensitive quality traits such as anthocyanin accumulation [3, 10, 37]. Vertical stacking maximizes production density [14], but can introduce microclimatic heterogeneity, requiring careful monitoring of airflow and temperature gradients to ensure uniform pigment accumulation [96]. Energy demand, particularly under low-temperature regimes, remains a major constraint, necessitating strategies that balance biological effectiveness with economic and operational feasibility [97, 98].

### HVAC-based and temporal temperature strategies

Effective temperature manipulation in plant factories depends on flexible HVAC systems capable of responding to species- and stage-specific thermal requirements [95, 99]. Zoning and variable refrigerant flow (VRF) systems allow facilities to operate multiple temperature regimes simultaneously, supporting tailored management across crops and developmental stages [100, 101]. Optimized airflow and humidity control further stabilize canopy microclimates and promote secondary metabolite synthesis while limiting disease pressure [102, 103]. A comparative summary of these temperature regulation strategies and their implications for anthocyanin enhancement is presented in Table 2.

**Table 2:** Temperature regulation strategies in plant factories and their implications for anthocyanin enhancement.

**Strategy**	**Temperature pattern**	**Primary advantages**	**Limitations**	**Suitable crops/stages**	**References**
Continuous HVAC cooling	Constant low setpoint	Stable pigment induction; high reproducibility	High energy demand; growth suppression if prolonged	Leafy greens; short production cycles	[7, 73]
Zoned*/*VRF HVAC control	Spatially differentiated temperatures	Crop- and stage-specific control; improved efficiency	Higher system complexity and cost	Mixed-species facilities	[93, 95]
Night-time cooling	Reduced night temperature only	Enhances anthocyanins with minimal biomass loss; energy-efficient	Requires precise timing and species calibration	Lettuce, red cabbage, purple potato	[104, 3]
Diurnal temperature modulation	Day-night temperature contrast	Synergistic effects on pigmentation and carbon balance	Risk of stress if gradients are excessive	Leafy greens, fruit, and vegetables	[3, 105]
Passive*/*assisted cooling	Thermal mass, night ventilation	Lower energy consumption; sustainability benefits	Climate-dependent effectiveness	Mild climates; hybrid systems	[106]

Beyond continuous thermal control, night-time cooling has emerged as a targeted and energy-efficient strategy for enhancing anthocyanin accumulation [107]. Cooler night temperatures stimulate anthocyanin biosynthetic gene expression and antioxidant capacity without severe growth reduction, particularly when applied in a species- and stage-specific manner [48, 108]. Energy-efficient approaches such as thermal mass storage and night ventilation can further reduce cooling demand while maintaining effective temperature differentials [109].

Collectively, these HVAC-based and temporal temperature strategies demonstrate that controlled, context-dependent cooling offers a practical pathway to enhance anthocyanin production while maintaining growth, energy efficiency, and commercial feasibility. Figure 3 provides a visual comparison of HVAC-based and night-time cooling strategies, highlighting key control mechanisms, advantages, and limitations for anthocyanin-oriented crop management. The bar chart further illustrates the relative responsiveness of representative crops to targeted temperature strategies, based on trends synthesized from the previous literature [3, 108].

**Figure 3:**
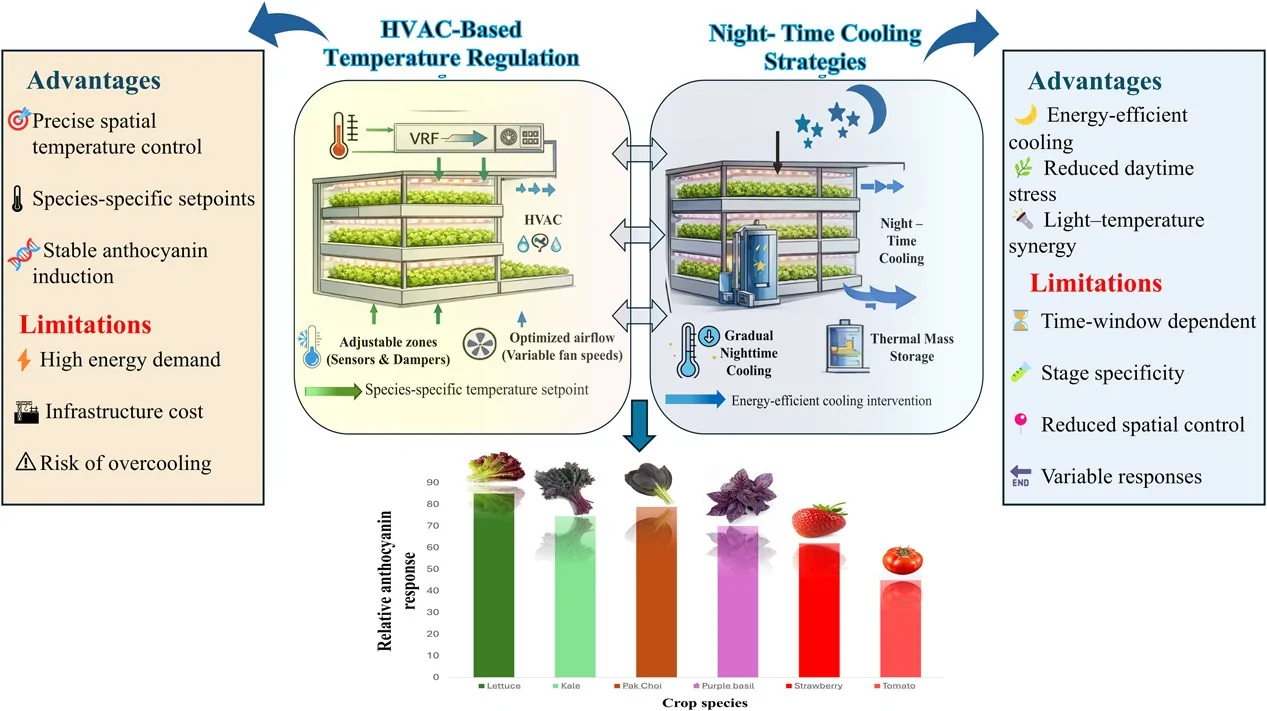
Overview of HVAC-based and night-time cooling strategies used in plant factory systems, illustrating key temperature control mechanisms, associated advantages, and operational limitations for anthocyanin-focused crop management. The accompanying bar chart depicts relative anthocyanin responsiveness among representative crops, with values conceptually synthesized from published studies.

### Precision mitigation: vertical farming core advantage

Building on the HVAC-based and temporal temperature strategies described above, VF systems enable precise mitigation of the trade-off between anthocyanin accumulation and plant growth [110]. VF allows temporal and spatial regulation of anthocyanin biosynthesis; however, excessive or prolonged induction can impose metabolic costs and reduce yield [14, 44, 89]. This limitation can be overcome through optimized induction timing and organ-specific targeting, enabling enhanced metabolite production without compromising plant performance [96, 111]. Short-term preharvest treatments (3–5 days) are particularly effective [112]. In leafy greens (e.g. lettuce), low-temperature exposure (12°C–15°C) during late development (days 26–30) increases anthocyanin levels by 3.3–3.9-fold, with only moderate yield reductions (7%–13%) [113, 114]. In fruit crops, night-time cooling further minimizes vegetative impacts by temporally separating metabolic induction from biomass accumulation [115].

Moreover, organ-specific targeting, such as leaves, inflores cence curds (e.g. cauliflower heads), or fruit epidermis, maximizes anthocyanin accumulation while avoiding whole-plant stress, thereby preserving overall productivity [4, 46]. Mechanistically, late-stage induction reduces competition for carbon and limits ROS-associated stress during active growth [48, 107]. Collectively, these strategies highlight that VF systems not only enhance anthocyanin enrichment but also provide a practical framework to minimize growth penalties through precise environmental control, supporting commercially viable production of high-value crops.

## Decision-driven temperature management

Building on plant factory platforms, DDTM operationalizes molecular insights into Part 1 to guide species-, stage-, and energy-aware preharvest cooling protocols. DDTM integrates baseline anthocyanin levels, growth stage, and operational constraints into actionable decisions, enabling consistent, high-quality anthocyanin enrichment while minimizing energy expenditure and avoiding growth reduction [30]. Importantly, DDTM incorporates quantitative performance indicators, including anthocyanin accumulation (mg·g^−1^ FW), energy consumption (kWh·m^−2^·cycle), and economic return, thereby strengthening its practical applicability in VF systems [116, 117]. By linking genotype-specific responses to precise environmental control, DDTM demonstrates how VF systems can translate molecular regulation into practical production strategies.

### D‌DTM decision framework

The DDTM framework defines critical decision parameters for VF production, including crop species, growth stage, baseline anthocyanin levels, and energy costs [44]. Activation of DDTM protocols depends on species-specific thermal sensitivity and developmental stage, ensuring that cooling interventions optimize anthocyanin accumulation without compromising plant growth or energy efficiency [118]. In addition, energy cost thresholds are explicitly incorporated to balance metabolic enhancement with operational feasibility, recognizing that energy expenditure represents a major component of VF production systems [97, 119]. These parameters are summarized in Table 3, which provides actionable thresholds and target actions for leafy greens, Solanaceae, and Rosaceae crops.

**Table 3:** DDTM decision parameters.

**Decision parameter**	**Thresholds**	**Action**
Crop species: leafy greens	15°C–20°C	Initiate cooling
Crop species: Solanaceae	12°C–15°C	Initiate cooling
Crop species: Rosaceae	12°C–18°C (night)	Initiate cooling
Growth stage	Days 25–32	Initiate cooling
Baseline anthocyanin	<2 mg·g^−1^ FW	Activate DDTM
Energy cost	<€0.12/kWh	Full protocol

Critical decision parameters for preharvest cooling in VF systems, adapted from controlled-environment horticulture, energy use studies, and cold-induced anthocyanin accumulation literature [3, 95, 120]. Energy thresholds are derived from reported ranges in controlled-environment and VF studies rather than illustrative assumptions [18, 119, 121].

### Case studies: red leaf lettuce and purple cauliflower

In red leaf lettuce, DDTM cooling was applied during the preharvest period (Days 26–30), returning to 20°C for the final 2 days to reduce growth suppression [20]. Molecular validation showed 3.2–5.1-fold upregulation of ICE1, CBF2, MYB75, and DFR, confirming activation of cold-mediated anthocyanin biosynthesis [64, 122]. The quantitative results are presented in Table 4.

**Table 4:** DDTM outcomes in red leaf lettuce and purple cauliflower under different cooling treatments.

**Crop**	**Treatment**	**Temperature (°C)**	**Duration**	**Anthocyanin (mg·g** ^**−1**^ **FW)**	**Yield (g/m** ^**2**^**)**	^**a**^ **Energy (kWh·m** ^**−2**^ **·cycle)**	**Net ROI ($·m** ^**−2**^**)**
Red lettuce	Control	22	–	1.2 ± 0.3	285 ± 12	45 ± 2	–
	DDTM-15	15	5 days	4.7 ± 0.5 (3.9×)	265 ± 15 (−7%)	58 ± 3 (+ 29%)	+ 2.28
	DDTM-12	12	3 days	3.9 ± 0.4 (3.3×)	248 ± 18 (−13%)	52 ± 2 (+ 16%)	+ 3.12
Purple cauliflower	Control	22	–	2.1 ± 0.2	750 ± 30	60 ± 3	–
	DDTM-14	14	5 days	5.6 ± 0.6 (2.7×)	710 ± 35 (−5%)	75 ± 4 (+ 25%)	+ 2.97
	DDTM-12	12	3 days	4.9 ± 0.5 (2.3×)	690 ± 40 (−8%)	70 ± 3 (+ 17%)	+ 4.23

a Energy consumption values are derived from VF studies [119, 123, 124]. Net ROI was calculated as the difference between anthocyanin value gain and additional energy cost (based on an electricity price of $0.12 kWh^−1^), using reported premium market values for anthocyanin-enriched crops [125]*.*

In purple cauliflower, DDTM was applied during head enlargement using short-term low-temperature exposure (14°C, Days 28–32) [21]. This treatment increased anthocyanin concentration by 2.3–2.7-fold relative to controls [21, 73], with only a slight yield reduction (−5% to −8%) and a moderate increase in energy costs. The detailed results are shown in Table 4.

Together, these case studies show that preharvest cooling guided by molecular and developmental cues can increase anthocyanin accumulation while limiting yield loss and energy use. The DDTM framework demonstrates how VF can integrate molecular regulation, developmental timing, and operational constraints into an actionable strategy for optimizing secondary metabolite production.

## Species-specific responses to cold: evidence from major horticultural crops

Cold-induced anthocyanin accumulation is species- and cultivar-dependent, reflecting differences in genetic regulation, metabolism, developmental sensitivity, and intrinsic cold tolerance [31, 126]. Responses vary across organs, developmental stages, and exposure durations, making cold stress a nonuniversal trigger for anthocyanin biosynthesis [45, 113]. This section summarizes effective temperature ranges for induction, associated physiological and yield trade-offs, and key knowledge gaps limiting reproducibility in controlled environments, with comparative data presented in Table 5.

**Table 5:** Species- and cultivar-specific low-temperature treatments for anthocyanin enhancement in horticultural crops under controlled-environment conditions.

**Crop group & examples**	**Temperature range (°C)**	**Developmental stage**	**Effects on anthocyanin**	**Growth/physiological trade-offs**	**Recommended plant factory strategy**	**References**
Leafy greens: red-leaf lettuce, Kale, radish sprouts	15–20	Vegetative	Moderate–high induction	Minimal growth penalty	Short-term preharvest or targeted cooling; avoid prolonged chilling	[20, 73, 114]
	10–12	Vegetative	High induction	Moderate growth suppression, delayed leaf expansion	Pre-harvest pulse cooling; monitor leaf area and fresh weight	[6, 113, 114]
	<10	Vegetative	Potentially high induction	Severe growth inhibition, chlorosis	Not recommended; risk of yield loss	[6]
Fruit vegetables (Solanaceae): tomato, pepper, eggplant	12–15	Vegetative/early fruit	Mild induction in specific tissues	Minimal in short term; tissue-specific	Short-term, light-coupled chilling; cultivar-specific	[36, 115]
	<10	Vegetative/early fruit	Limited induction	Growth suppression, delayed flowering, reduced fruit set	Avoid prolonged exposure	[51]
Berries/Rosaceous crops: strawberry, apple	12–18 (night)	Fruit ripening	Moderate induction; enhanced color and antioxidant	Minimal if timed properly	Cool night temperature during late ripening; monitor sugar and aroma	[127, 128]
	<10	Fruit ripening	Variable induction	Sugar reduction, flavor impairment	Use with caution; cultivar-specific	[48]
Other horticultural crops: grapevine, purple broccoli	10–15 (diurnal fluctuation)	Vegetative	Moderate–high induction	Low to moderate	Day/night temperature modulation; monitor leaf photosynthesis	[96, 129]

### Leafy greens: red mustard, kale, and radish sprouts

Leafy vegetables, particularly Brassicaceae crops such as red mustard (*Brassica juncea*), kale, Chinese cabbage (*Brassica rapa* ssp. *pekinensis*), pak choi (*Brassica rapa* ssp. *chinensis*), and radish sprouts, exhibit rapid anthocyanin induction under mild chilling [3, 36, 112]. In red mustard and pak choi, temperatures around 10°C–18°C promote anthocyanin accumulation and antioxidant capacity through activation of flavonoid biosynthetic pathways, whereas exposure below 8°C–10°C can lead to growth inhibition, chlorosis, and delayed leaf expansion [112, 130]. Kale responds similarly to moderate chilling (10°C–15°C), showing enhanced anthocyanin content and antioxidant activity, while prolonged exposure below 8°C–10°C decreases growth rates and photosynthetic efficiency [112].

Short-term cold exposure in radish sprouts and other leafy Brassica crops enhances pigmentation and antioxidant activity, but extended chilling impairs biomass accumulation [32]. Cultivar-dependent variation is significant; red- or purple-pigmented Brassica genotypes generally exhibit stronger cold responsiveness than green cultivars due to higher basal expression and inducibility of anthocyanin-related MYB transcription factors [3, 6, 32]. Overall, leafy greens display low temperature thresholds for anthocyanin induction but limited tolerance before growth inhibition occurs, making them particularly suitable for short-term or preharvest chilling strategies in CEA.

### Fruit vegetables: tomato, eggplant, and pepper

Fruit vegetables of the Solanaceae family, including tomato (*Solanum lycopersicum*), pepper (*Capsicum annuum*), eggplant (*Solanum melongena*), and chili, exhibit more constrained and context-dependent anthocyanin responses [131, 132]. In tomato and pepper, low temperatures primarily activate cold-acclimation and stress-protection pathways, while anthocyanin accumulation remains limited, tissue-specific, or transient [133, 134]. For instance, tomato exposed to 12°C–15°C shows upregulation of anthocyanin-related genes; however, prolonged chilling below 10°C suppresses photosynthetic performance and fruit development [135].

Eggplant is comparatively more responsive, as mild chilling can induce early activation of anthocyanin biosynthetic genes and MYB transcription factors; nevertheless, extended exposure delays fruit maturation and reduces yield [136]. Collectively, these findings indicate that these cold-response mechanisms in Solanaceae fruit crops are not directly comparable to those in leafy vegetables, emphasizing the need for precise temporal and environmental control to achieve effective preharvest anthocyanin enhancement.

### Rosaceae fruit crops: strawberry, sweet cherry, and peach

Rosaceous fruit species, including strawberry (*Fragaria × ananassa*), sweet cherry (*Prunus avium*), and peach (*Prunus persica*), show moderate but commercially relevant anthocyanin responses to low temperatures during fruit ripening [79, 137, 138]. In strawberry, cool night temperatures enhance fruit coloration via *MYB10* activation, improving antioxidant content and visual quality, whereas excessive chilling can reduce sugar accumulation and flavor quality [79].

In sweet cherry, cool temperatures promote skin pigmentation through activation of anthocyanin-related regulatory genes such as *MYB*, enhancing fruit quality [138]. Peach cultivars show strong genotype-dependent variation, where cool night temperatures can enhance peel coloration, although responses differ with developmental stage and environmental conditions [137]. Taken together, anthocyanin accumulation in Rosaceae fruits is tightly regulated by genotype, development, and temperature, requiring precise environmental management under controlled conditions.

### Research gaps and practical implications for plant factories

Despite increasing attention, significant uncertainties remain due to variability in temperature regimes, exposure durations, developmental stages, and response metrics [3, 91]. Most studies emphasize short-term anthocyanin enhancement without systematically evaluating effects on growth, yield, or postharvest quality [96, 136]. Critical thresholds distinguishing beneficial pigment induction from physiological stress or damage remain poorly defined.

Addressing these gaps requires species- and cultivar-specific response mapping, cross-crop comparisons, and integration with controlled-environment climate strategies. Plant factories, which enable precise regulation of temperature intensity, timing, and duration, provide an ideal platform to align cold treatments with physiological thresholds [14, 36]. Such approaches can optimize anthocyanin accumulation while minimizing growth penalties, thereby supporting energy-efficient production of nutritionally enhanced crops [14, 37].

## Trade-offs, risks, and practical constraints of cold-induced anthocyanin enhancement

### Physiological stress and growth trade-offs

Cold stress redirects plant metabolism from growth to survival, prioritizing stress tolerance over biomass accumulation [54]. Chilling-sensitive crops such as tomato, pepper, and eggplant exhibit chlorosis, photoinhibition, reduced photosynthesis, and decreased fruit set under low temperatures [115]. Even relatively tolerant crops, including red-leaf lettuce, kale, and radish sprouts, may show transient growth suppression [3, 7, 112]. These responses indicate that anthocyanin enhancement is not universally beneficial and depends on species-specific thermal thresholds.

Growth suppression is associated with ROS accumulation, activation of CBF–COR signaling, hormonal modulation (ABA, cytokinins, gibberellins), and sugar-mediated pathways, which collectively redirect carbon and energy toward secondary metabolism at the expense of biomass accumulation [4, 57]. In fruiting crops, chilling during vegetative or early reproductive stages may delay flowering and reduce fruit set rather than enhance pigmentation [48, 118]. Cold injury can also impair respiration, membrane stability, photoprotection, and nutrient uptake, highlighting the need to balance stress induction with growth maintenance [38].

### Operational and energy constraints

Cold treatments in plant factories are constrained by HVAC demand, energy costs, and microclimate heterogeneity across vertical layers, which results in uneven pigmentation and growth [98, 100]. Targeted strategies, such as short-term preharvest chilling, night-time temperature reduction, or localized cooling, can enhance anthocyanin accumulation while minimizing energy use [46]. These approaches require precise timing, crop-specific thresholds, and sensitivity to developmental stage [9, 18, 32]. Integration of environmental sensors, predictive modeling, and automated climate control enables spatial and temporal precision, reducing overexposure and operational inefficiencies [32, 89]. Strategic scheduling, including pulse or intermittent cooling, can further limit cumulative stress and energy consumption [97]. For example, late-stage chilling in blood orange enhances pigmentation without compromising fruit development when aligned with active anthocyanin biosynthesis [81].

### Nutritional and postharvest quality

Cold-induced anthocyanins improve visual quality and antioxidant capacity; however, their stability and bioavailability depend on factors such as glycosylation, acylation, pH, co-pigmentation, and metabolite interactions [3, 107]. Excessive chilling may accelerate senescence, disrupt tissue integrity, and reduce pigment stability and antioxidant activity [113, 126]. In fruit crops such as strawberry and apple, improper temperature management can also diminish sugar content, flavor, and overall quality [27, 31]. Short-term, late-stage, or pulse chilling treatments can maximize anthocyanin accumulation while preserving metabolic balance, flavor, and postharvest performance [115]. Integrating species-specific physiology, developmental timing, and environmental control is therefore essential to ensure that cold-induced pigmentation enhances nutritional value without compromising yield, shelf life, or commercial viability [52].

## Translating mechanisms into practice: conditional temperature strategies in plant factories

Mechanistic insights into cold-induced anthocyanin biosynthesis can be translated into plant factory applications through conditional temperature control, genotype × environment ×  management (G × E × M) optimization, sensor- and AI-assisted monitoring, and microbiome-based modulation [3, 89, 139]. Together, these strategies enable enhanced anthocyanin accumulation while maintaining crop productivity, energy efficiency, and operational feasibility in controlled-environment systems.

### Conditional temperature management

Cold treatments should be applied selectively, considering species, cultivar, developmental stage, and production objectives [4, 37]. In leafy vegetables, short preharvest chilling activates MBW regulators and key biosynthetic genes (*PAL, CHS, DFR, ANS*), enhancing pigmentation without prolonged growth reduction [32]. In contrast, fruit vegetables (e.g. pepper, cucumber) often require combined cues such as chilling with high light or UV-enriched spectra [21, 134], while berries respond most effectively to cool night temperatures during late ripening [79, 127]. Short-term or pulse chilling leverages the rapid responsiveness of anthocyanin pathways while avoiding sustained stress, enabling efficient pigment enhancement within defined developmental windows [32, 51].

### Integrating genotype × environment (G × E) interactions

Anthocyanin responses to low temperature are strongly genotype-dependent, reflecting variation in MBW regulation, CBF–COR signaling, and associated metabolic pathways [4, 56]. Even within a species, cultivars differ in temperature sensitivity, response kinetics, and pigment accumulation capacity [140]. For example, lettuce cultivars vary in chilling requirements for optimal pigmentation, while some kale genotypes exhibit enhanced stress tolerance that may limit carbon allocation to secondary metabolism [6, 112]. Environmental factors, including light quality, irradiance, photoperiod, CO₂ concentration, and nutrient availability, interact with genotype to shape anthocyanin responses [3]. High-intensity or blue/UV-enriched light can enhance pigmentation, whereas nutrient or carbon limitations may constrain biosynthesis [12, 41]. Effective implementation, therefore, requires genotype-specific response frameworks defining optimal temperature regimes, exposure duration, and environmental compatibility.

### Sensor-based monitoring, digital twins, and AI models 

Advanced sensing technologies form the backbone of precision anthocyanin management in plant factories by enabling real-time assessment of plant physiological status [89, 139, 141]. Hyperspectral, multispectral, near-infrared, and Raman imaging provide nondestructive estimation of anthocyanin content alongside carbohydrates, proteins, carotenoids, and sugars [3, 96]. When combined with electrophysiological measurements and chlorophyll fluorescence, these tools capture dynamic stress responses, photosynthetic efficiency, and metabolic shifts associated with chilling treatments [41, 141]. Together, these data streams support predictive modeling of pigment accumulation under variable environmental conditions.

Digital twins integrate sensor data with mechanistic models to simulate crop, environment interactions and predict responses to temperature, light, and CO₂ manipulation [89]. AI-assisted decision-support systems, including reinforcement learning frameworks, use these simulations to recommend conditional interventions such as preharvest pulse chilling or spatially localized cooling, reducing unnecessary stress and energy expenditure [139]. Importantly, these tools function as decision aids rather than fully autonomous controllers, allowing growers to apply expert judgment while benefiting from data-driven precision.

### Probiotics and microbiome considerations

The plant-associated microbiome represents an emerging strategy for modulating anthocyanin biosynthesis under cold stress [142]. Beneficial microorganisms, including *Bacillus*, *Pseudomonas*, *Azospirillum*, and *Trichoderma* species, enhance nutrient uptake, hormone signaling, and stress tolerance, indirectly supporting phenylpropanoid metabolism [143]. Some plant growth-promoting rhizobacteria (PGPR) strains also improve sugar availability and antioxidant capacity, creating favorable conditions for anthocyanin accumulation during chilling [144].

Microbiome-based approaches may further reduce growth inhibition by improving stress resilience and metabolic stability, particularly in chilling-sensitive crops [144]. Effective implementation requires consideration of strain specificity and environmental interactions. Combined with targeted temperature and light strategies, these approaches support efficient pigmentation enhancement without compromising productivity.

## Emerging tools, research needs, and priority directions

Temperature-based modulation of anthocyanin biosynthesis represents a promising strategy for quality-oriented crop management in CEA; however, its practical implementation remains constrained by unresolved biological variability and system-level complexity. As illustrated in Fig. 4, effective translation requires tighter integration of plant physiology, sensing technologies, and environmental control engineering. This section synthesizes key limitations and priority research directions across three interconnected domains: species- and cultivar-specific responses, emerging precision-management tools, and standardized, hypothesis-driven research frameworks.

**Figure 4:**
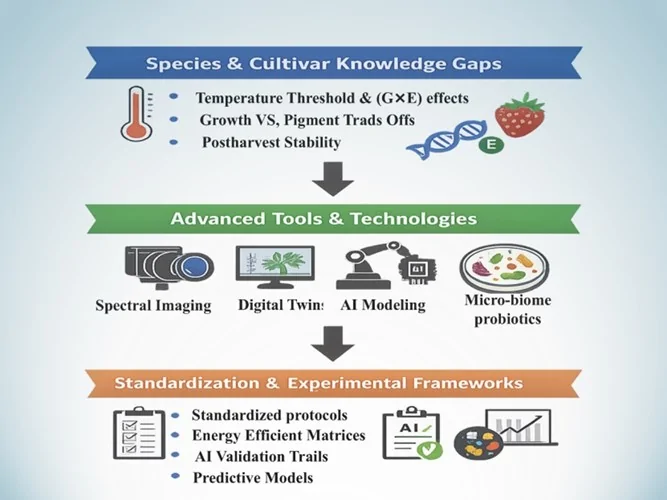
Conceptual framework linking knowledge gaps, emerging tools, and priority research directions for cold-induced anthocyanin enhancement in plant factories. The framework integrates species- and cultivar-specific responses, precision tools, and standardized performance metrics to support reproducible and energy-efficient strategies in controlled environments.

### Species- and cultivar-specific research needs

A major limitation in applying cold-induced anthocyanin strategies is the incomplete understanding of species- and cultivar-specific responses [145]. Most studies emphasize short-term pigmentation outcomes in single crops, with limited evaluation of growth performance, yield, and postharvest quality [11, 36]. Comparative evidence suggests that leafy greens (e.g. red-leaf lettuce) tolerate mild chilling, whereas fruit crops (e.g. tomato, pepper, strawberry) have narrower temperature tolerance ranges, beyond which chilling induces stress rather than enhancing pigmentation [3, 45]. Developmental stage, source–sink dynamics, and interactions with light further modulate these responses but remain insufficiently resolved across species [37, 41].

Addressing these gaps requires systematic, crop-specific studies that quantify temperature thresholds and exposure durations to maximize anthocyanin accumulation while minimizing constraints on biomass production and fruit quality. Future research should resolve genotype × environment (G × E) interactions involving light, nutrients, and photoperiod, and evaluate postharvest stability and bioavailability, including glycosylation, acylation, pH effects, and co-pigmentation. Such efforts will enable stage-specific, cultivar-optimized temperature strategies that balance pigmentation, physiological performance, and commercial feasibility.

### Integration of emerging tools for precision control

Emerging sensing and modelling technologies increasingly bridge mechanistic understanding with operational control. Nondestructive tools, including hyperspectral, multispectral, near-infrared, and Raman spectroscopy, alongside chlorophyll fluorescence and thermal monitoring, enable real-time assessment of anthocyanins and stress status [141]. When integrated with digital twins and AI-driven predictive models, these systems can simulate environmental scenarios, forecast pigment responses, and support conditional, closed-loop temperature interventions in plant factories [89].

Complementary approaches such as microbiome modulation and probiotic applications offer additional leverage by enhancing stress tolerance and secondary metabolism without increasing thermal load [142, 144]. Although validation remains limited, combining microbial strategies with targeted chilling, pulse cooling, or zone-specific temperature control could improve anthocyanin enrichment while reducing energy consumption and maintaining crop vigor in high-density systems.

### Standardization, experimental frameworks, and hypothesis-driven research

Translation to commercial plant factory systems is constrained by inconsistent experimental designs and reporting metrics, which limit cross-study comparability and predictive modelling [89, 93]. Standardized frameworks must capture key dimensions of temperature-mediated pigmentation, including cumulative and pulse temperature exposure relative to developmental stage, anthocyanin yield per unit energy input, growth–quality trade-offs under controlled environments [36, 97], and postharvest stability and bioavailability.

Future research should shift from descriptive stress-response studies toward hypothesis-driven designs that integrate mechanistic insights with controlled-environment validation. Priority directions include: defining cultivar-specific temperature response ranges and associated trade-offs; validating AI-assisted decision-support tools in operational plant factories; evaluating microbiome-based interventions for pigment stability and stress resilience; and developing harmonized reporting protocols linking environmental inputs with anthocyanin outcomes, energy efficiency, and postharvest quality. Collectively, these advances will support predictive, scalable, and nutritionally relevant anthocyanin management in next-generation plant factory systems.

## Conclusion

Based on this review, the following conclusions can be drawn: (i) cold-induced anthocyanin accumulation represents an effective although highly conditional strategy for enhancing the nutritional, functional, and sensory quality of horticultural crops in plant factories; (ii) temperature functions not as a uniformly beneficial stress but as a regulatory signal whose outcomes depend on species, cultivar, developmental stage, and interactions with light environment, nutrient availability, and carbon allocation; (iii) strategically timed or short-term preharvest chilling can enhance pigmentation and antioxidant capacity while minimizing growth limitations, whereas prolonged or excessive cold exposure often induces physiological stress and reduces production efficiency; and (iv) plant factories provide an ideal platform for implementing such targeted temperature strategies due to their high controllability and reproducibility, particularly when combined with advanced non-destructive sensing, AI-assisted predictive models, and digital twins that enable closed-loop environmental control. Emerging strategies such as microbiome modulation may further enhance stress resilience and anthocyanin biosynthesis, although further validation is required. Finally, standardized metrics and reporting frameworks are essential to ensure reproducibility, food safety, and scalable implementation.
